# Multiple Clinical Indications of Mifepristone: A Systematic Review

**DOI:** 10.7759/cureus.48372

**Published:** 2023-11-06

**Authors:** Sharon Mathew, Maria S Ticsa, Soniya Qadir, Aida Rezene, Deepesh Khanna

**Affiliations:** 1 Foundational Sciences, Nova Southeastern University Dr. Kiran C. Patel College of Osteopathic Medicine, Clearwater, USA; 2 Medical School, Nova Southeastern University Dr. Kiran C. Patel College of Osteopathic Medicine, Clearwater, USA; 3 Osteopathic Medicine, Nova Southeastern University Dr. Kiran C. Patel College of Osteopathic Medicine, Clearwater, USA

**Keywords:** uses of mifepristone, ptsd, psychotic depression, leiomyomas, post-coital contraception, abnormal uterine bleeding, gynecologic and obstetric conditions, abortion pill online, synthetic prostaglandin e1 analog, mifepristone

## Abstract

Mifepristone and misoprostol are globally used medications that have become disparaged through the stigmatization of reproductive healthcare. Patients are hindered from receiving prompt treatment in clinical scenarios where misoprostol and mifepristone are the drugs of choice. It is no exaggeration to emphasize that in cases where reproductive healthcare is concerned. The aim of this paper is to discuss the different indications of mifepristone and to delineate where the discrepancy in accessibility arises. For this systematic review, we included publications citing clinical trials involving the use and efficacy of mifepristone published in English within the date range of 2000 to 2023. Five databases were searched to identify relevant sources. These databases are Google Scholar, MEDLINE with full text through EBSCO, and three National Center for Biotechnology Information (NCBI) databases (NCBI Bookshelf, PubMed, and PubMed Central). Twenty-three records were ultimately included in this review. Mifepristone has been shown to have therapeutic effects in the treatment of psychiatric disorders, such as major depressive disorder and psychotic depression. There was a significant decrease in depression and psychiatric rating symptoms for patients taking mifepristone versus placebo with no adverse events. Mifepristone has also been shown to improve treatment course in patients with Cushing’s disease (CD) who failed or are unable to undergo surgical treatment. In addition, mifepristone has been shown to be a successful treatment option for adenomyosis and leiomyomas. Patients had a statistically significant decrease in uterine volumes following mifepristone treatment, which aided in the alleviation of other symptoms, such as blood loss and pelvic discomfort. Mifepristone is a synthetic steroid that has immense potential to provide symptomatic relief in patients suffering from a wide array of complicated diseases. Historically, mifepristone has been proven to have an incredible safety profile. While further research is certainly needed, the politicization of its medical use for only one of its many indications has unfortunately led to the willful ignorance of its potential despite its evidence-based safety profile and efficacy.

## Introduction and background

Mifepristone is a synthetic steroid derived from norethindrone and therefore has antagonistic activity against progesterone and glucocorticoid receptors. Misoprostol is a synthetic prostaglandin E1 analog that works through the direct stimulation of prostaglandin E1 receptors. Recently, these medications have become disparaged due to their associations with the controversial medical procedure known as abortion. Abortions, however, have been so common that one out of four women will have had an abortion by the time they reach the age of 45 [1]. It is estimated that 3.7 million women have used mifepristone and misoprostol for medication abortions since they were first approved by the Food and Drug Administration (FDA) in 2000 [1]. Mifepristone followed by misoprostol is up to 14 times safer than carrying the patient’s pregnancy to term [1]. Aside from abortion, mifepristone is used for both gynecologic and obstetric conditions. Obstetric conditions include induction of labor, postpartum hemorrhage, intrauterine fetal demise, ectopic pregnancies, and miscarriages [2]. Gynecological conditions that can be treated with mifepristone include abnormal uterine bleeding, post-coital contraception, and treatment of gynecological cancers [3]. Due to the stigmatized nature of abortion, however, patients are hindered from receiving prompt treatment in clinical scenarios where mifepristone is the drug of choice. It is no exaggeration to emphasize that in cases where reproductive healthcare is concerned, every second counts [3]. Legislation that varies across states further impacts patients who risk their lives and health as they attempt to navigate their care plan across borders. Travel costs, time-off, childcare, transportation, and living accommodations are just a few more of the factors patients must take into consideration when they are forced to seek life-saving care outside of their homes [3].

Mifepristone is a medication that has multiple therapeutic applications, such as treating leiomyomas, psychotic depression, and post-traumatic stress disorder (PTSD). However, its use is restricted in many countries because of its abortifacient effect. This is a logical fallacy that deprives patients of a beneficial and safe treatment option. This systematic review aims to explore the evidence-based uses of mifepristone and how it can improve patients' health outcomes. The clinical indications that will be discussed are adenomyosis, leiomyomas, psychotic depression, PTSD, and Cushing's disease (CD).

## Review

Methods

Eligibility Criteria

For this systematic review, we included publications of clinical trials and systematic reviews citing clinical trials relating to the clinical use of mifepristone and published in English within the date range of 2000 to 2023.

Info Sources

Five databases were searched to identify relevant sources. These databases include Google Scholar, MEDLINE with full text through EBSCO, and three National Center for Biotechnology Information (NCBI) databases (NCBI Bookshelf, PubMed, and PubMed Central).

Search Strategy

For each database, we inputted “clinical use of mifepristone” as our search term. The populated results were then narrowed down to those published in the English language and within the date range of 2000 to 2023 using automated search tools. 

Selection Process

The titles and abstracts of the remaining records were then screened, and those deemed relevant to clinical uses of mifepristone and its efficacy were included for comprehensive review. This initial record search in three of the four databases (Google Scholar, MEDLINE, and PubMed) was completed by three separate reviewers. The initial record search in the remaining two databases (NCBI Bookshelf and PubMed Central) was completed by another individual reviewer.

Data Collection Process

After the initial record search, 60 records were deemed relevant to the study topic and compiled for a more comprehensive review. Two records were found to be duplicates and removed. Each of the four reviewers read the remaining 58 records and voted on the eligibility of the publication for inclusion in our review. Older publications that were expanded upon in more recent study trials were excluded to reduce redundancy. In addition, for records with similar study protocols, only the more recently published record was included. Ten records were excluded from the review due to ineligible study design. For those records that were not unanimously accepted (at least one reviewer voted for exclusion), the record was excluded. To ensure that the data utilized in this review were backed by sufficient evidence, the reviewers organized the remaining records into groups based on the disease mifepristone was being studied to treat. After further discussion, it was decided to exclude the records in the groups that lacked at least three separate clinical trials on the use of mifepristone in the treatment of the disease. Thirty articles were excluded. Seven of the 18 remaining records were systematic reviews, and citation searching of the records found four additional records that met the eligibility criteria. The remaining 23 records were included for further review.

Data Items

Of the remaining 23 records deemed acceptable for inclusion, only studies with statistically significant findings regarding the clinical use of mifepristone were included for detailed analysis. One record was excluded due to early termination of the trial. Our records include two open-label studies, four retrospective studies, seven reviews (systematic, meta-analysis), one wet lab (human specimen was used), five long-term safety extension articles, and seven randomized control experimental trials.

Study Risk-of-Bias Assessment

We assessed the risk of bias (RoB) in the studies included in the review using the revised Cochrane RoB tool for randomized trials (RoB 2). The five domains assessed were (1) RoB arising from the randomization process, (2) RoB due to deviations from the intended interventions (effect of assignment to intervention and effect of adhering to intervention), (3) missing outcome data, (4) RoB in the measurement of the outcome, and (5) RoB in the selection of the reported result. Each randomized control trial included in this review was assessed for RoB by two authors working independently using the RoB 2. For those studies in which the assessing authors came to different conclusions, the remaining two authors completed independent RoB 2 assessments of the study in question, and the majority of findings was accepted. Utilizing the methodology for assigning the overall RoB for each study as outlined by the RoB 2 tool, each study was designated as having “low risk of bias” or “high risk of bias.” After an initial assessment, both authors deemed the nine randomized control studies had a low RoB.

Effect Measures

Analysis of the studies included a focus on statistically significant findings that varied between control and intervention groups as defined by a p-value less than 0.5. As each study had its own parameters and primary and secondary endpoints, we focused our analysis on the safety and clinical efficacy of mifepristone as measured and reported by the authors of the studies included.

Synthesis Methods

As previously mentioned, as the studies included in this review vary widely in their study population and intervention design, our analysis focused on qualitative synthesis of study outcomes. These outcomes were categorized as the clinical efficacy and safety of mifepristone for CD, psychiatric disorders, and select gynecological diseases (adenomyosis and leiomyomas).

Certainty Assessment

To assess the certainty of the body of evidence regarding the studies included in our review, two reviewers applied the five Grading of Recommendations, Assessment, Development, and Evaluations (GRADE) considerations (study limitations, inconsistency of results, indirectness of evidence, imprecision, and publication bias) to each study. Accordingly, the included studies were categorized as having high, moderate, low, or very low certainty of evidence based on the GRADE criteria. After the assessment, both reviewers deemed that all records had high certainty of evidence.

**Figure 1:**
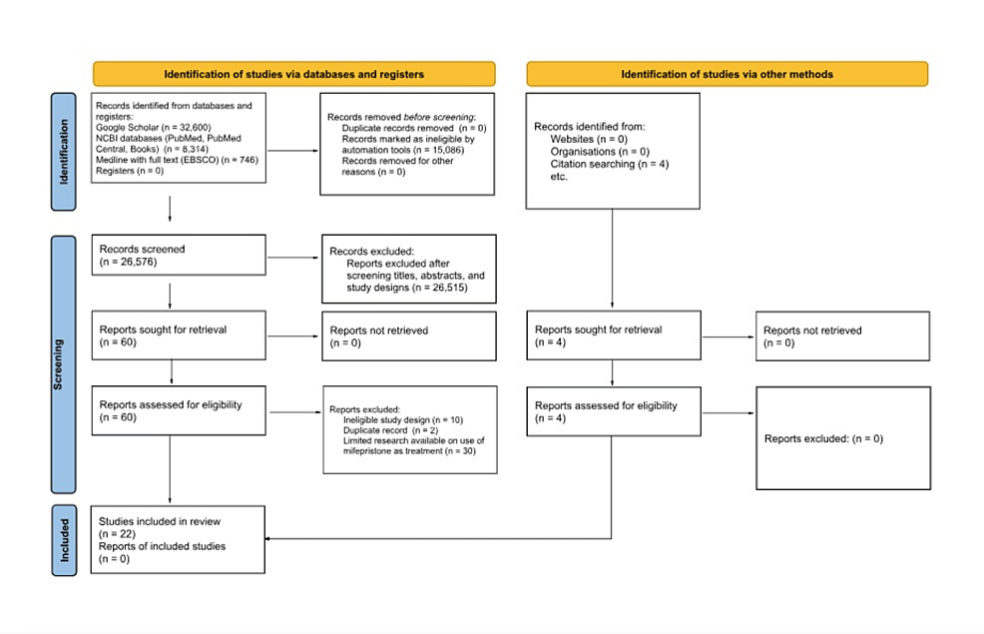
PRISMA 2020 flow diagram for new systematic reviews that included searches of databases, registers, and other sources *Consider, if feasible to do so, reporting the number of records identified from each database or register searched (rather than the total number across all databases/registers). **If automation tools were used, indicate how many records were excluded by a human and how many were excluded by automation tools. PRISMA: Preferred Reporting Items for Systematic Reviews and Meta-Analyses

Results

Psychiatric Implications

Based on the analyses, numerous trials demonstrated the profound therapeutic effect that mifepristone can have on psychiatric disorders. In a double-blind study following 19 patients with bipolar disorder, researchers studied neurocognitive function and mood in patients treated with mifepristone vs. the placebo [4]. Significant improvements in verbal fluency and spatial working memory were seen in the group treated with mifepristone. The Hamilton Depression Rating Scale (HDRS) and Montgomery-Asberg Depression Rating Scale (MADRS) scores also improved from baseline (i.e., lower scores) measurements in these patients. It is worth noting that these improvements were seen in as little as two weeks, which is quicker than what is normally seen with typical therapeutic agents for bipolar disorder (lithium/valproic acid) [4].

The most extensive research demonstrated the benefits of using mifepristone with major or psychotic depression [5]. It is important to note that approximately 20% of patients living with major depression experience psychotic symptoms [6]. A randomized, double-blind study looked at 30 participants with psychotic major depression (PMD) and treated them with mifepristone 600 mg or a placebo for eight days. Using the HDRS and Brief Psychiatric Rating Scale (BPRS) to quantify baseline levels of symptoms, results from eight days later showed that mifepristone was significantly more effective in reducing psychotic symptoms compared to the placebo group [6]. By day 8, nearly half of the participants attained a 50% reduction in the BPRS compared to the placebo group (p<0.046) in addition to lower HDRS scores (although this was not found to be significant). Moreover, when researchers looked further into the use of mifepristone in psychotic depression disorders, they discovered a correlation between higher plasma levels of mifepristone and a reduction in psychotic symptoms [7]. More specifically, the strongest reduction in psychosis symptoms was found to be associated with doses of 1200 mg/day of mifepristone, which resulted in a statistically significant reduction in psychotic symptoms (p<0.0004) [7]. The drug was also well tolerated and demonstrated a large safety margin in contrast to the numerous common adverse effects that patients experience when placed on standard treatment options (i.e., antipsychotics). In another double-blind, placebo-controlled study that took place over four days, five participants diagnosed with psychotic major depression were administered 600 mg of mifepristone [5]. The HDRS and BPRS scores were used, and the results showed that all five participants' depression ratings decreased - a nearly statistically significant finding (p<.07) [5]. Likewise, four out of the five BPRS scores declined, approximating to a 32.5% decline, which is comparable to the 40% decline seen with traditional antipsychotic treatments that span six to eight weeks. Once again, no adverse effects were reported.

The use of mifepristone has been explored in many cognitive disorders, including Alzheimer's disease. One study found that patients with mild to moderate Alzheimer’s disease displayed improvement on the Alzheimer’s disease assessment cognitive subtest - by 2.67 as opposed to the 1.67 decline in patients treated with a placebo [5]. Although not statistically significant, this finding encourages further studies to continue exploring the psychiatric and neurologic use of mifepristone.

Cushing’s Disease

Multiple trials have been conducted regarding the use and efficacy of mifepristone in the treatment of CD. Although surgical intervention to remove the source of excess cortisol production is the current mainstay of treatment, clinical trials have focused on the treatment with mifepristone for medical therapy, especially in patients who have failed surgical intervention or for those who are not good candidates for surgery.

Accordingly, a retrospective study of 20 patients with hypercortisolism (12 with adrenocortical carcinoma, three with ectopic adrenocorticotropic hormone (ACTH) secretion, four with CD, and one with bilateral adrenal hyperplasia) found clinically significant improvement in excess cortisol-induced symptoms in 15 out of 20 patients [4]. Patient responses to mifepristone treatment were monitored by clinical signs of hypercortisolism (signs of hypercortisolism, blood pressure measurements, and signs of adrenal insufficiency) and serum potassium and glucose. The study found that 15 out of 20 patients showed significant clinical improvement in excess cortisol-induced symptoms. Psychiatric symptoms and blood glucose levels also improved in the patients [4]. Of note, 11 out of 20 trial participants exhibited moderate to severe hypokalemia as a side effect, although only one patient had to leave the study early due to severe adverse effects [4].

In another well-known study, 50 patients were assessed at baseline and during intervention (total of six times) for 24 weeks, referred to as the SEISMIC study [8]. Changes in oral glucose tolerance tests over time were used to assess the mifepristone effect in type 2 diabetes millets (T2DM)/impaired glucose tolerance patients. Changes in diastolic blood pressure (BP) over time were used to measure the effect of mifepristone in hypertensive cardiogenic shock (CS) patients [8]. Results found a statistically significant improvement in symptoms in both groups: diabetic patients had improvement in response to oral glucose test, decreased A1C, and decreased fasting glucose, and hypertensive patients had decreased diastolic BP or reduction in antihypertensive medications [8]. In addition, the waist circumference and hemoglobin A1C (HbA1C) also improved, and study findings concluded that mifepristone use has an acceptable risk-benefit ratio for six months of treatment [8].

Several extension studies were later performed utilizing the data found during the SEISMIC study [9]. One such study assessing weight loss in patients who participated in the SEISMIC study also found statistically significant improvement in patients with CD. After one-week mifepristone period (patients who chose to participate in this follow-up study had to be assessed to ensure it was safe for them to enroll in this study), 30 patients were enrolled and started on once daily mifepristone at the dose they were taking when the SEISMIC study concluded [9]. The patient's weight was assessed at baseline and week 24 of the SEISMIC study, and for this study, the follow-up weight was taken at months 6, 12, 18, and 24 and a final visit. Data were assessed for 29 of the participants and statistically significant decreases in weight were found for all participants from baseline to end of the SEISMIC study, and the maintenance of weight loss was statistically significant in all participants at their final visit to this study as well [9].

Another SEISMIC extension study focused on monitoring the effects of mifepristone treatment in CD on ACTH levels and pituitary MRI findings [10]. Serum ACTH, urinary, and salivary cortisol levels were monitored during the SEISMIC study (baseline, day 14, and weeks 6, 10, 16, and 24) and once after a six-week mifepristone-free "washout" period. ACTH levels were then monitored one month later and then routinely every three months during the intervention period, which varied per participant [10]. Serum cortisol measures were assessed during the SEISMIC study at the intervals mentioned previously and then every six months during the extension study. Pituitary MRI studies were taken prior to mifepristone administration during the SEISMIC study and at weeks 10 and 24 [10]. Repeat imaging was then taken every six months during the extension study. On average, ACTH levels increased greater than twofold (2.76 ± 1.65-fold over baseline; p<0.0001 vs. baseline) in patients during the SEISMIC and extension study periods and decreased to near baseline levels after six weeks of mifepristone discontinuation [10]. Serum cortisol levels in both the initial intervention and extension period increased as well, although a higher mean cortisol level was seen during the extension study intervention (SEISMIC: 1.97 ± 1.02-fold increase; p<0.0001 vs. baseline; extension study: 2.85 ± 1.05-fold increase; p<0.0001 vs. baseline) [10]. In comparing the baseline and post-intervention MRI images, 30 out of 36 patients showed no progression in pituitary tumor size with mifepristone intervention, two patients showed regression of tumor size, and three patients showed evidence of tumor progression. One patient was found to have a tumor post-intervention despite a negative initial MRI at baseline [10].

A retrospective analysis of data collected during the SEISMIC study utilized oral glucose tolerance test data to assess the mifepristone treatment effect on the total body insulin sensitivity, beta cell function, weight, waist circumference, and additional parameters [11]. The analysis found improved total body insulin sensitivity in all participants, with the greatest improvement occurring from baseline to week 6. The weight and waist circumference both decreased by week 24 [11].

An additional important six-month study was done on 46 patients with refractory CS and either DM2, impaired glucose tolerance, or diagnosis of HTN in which mifepristone treatment was administered daily [12]. Patients were examined by three separate reviewers using global clinical response assessments (-1 = worsening, 0 = no change, 1 = improving) measured by eight clinical categories: glucose control, lipids, blood pressure, body composition, clinical appearance, strength, psychiatric/cognitive symptoms, and quality of life at weeks 6, 10, 16, and 24. A positive correlation with increasing GCR scores was found by week 24, with 88% of participants showing statistically significant improvement (p<0.001) [12].

Adenomyosis/Leiomyoma

Adenomyosis and leiomyomas are common gynecological conditions that affect large portions of the female population. Multiple trials have proven mifepristone’s success in treating endometriosis and various forms of cancer. Current data shows that mifepristone is well tolerated and has mild side effects in certain long-term clinical settings.

In one trial following mifepristone and its effects on adenomyosis, 20 patients were treated with 5 mg oral mifepristone/day for three months [13]. After the three-month trial, patients demonstrated a statistically significant (p<0.001) reduction in uterine volume as was measured through transvaginal ultrasound. These patients were also found to have significantly decreased CA-125 markers (a marker of adenomyosis and an increase in uterine size) and significantly increased hemoglobin concentration The patient’s endometrial tissue was then obtained from each patient during their hysterectomy [13]. The endometrial tissue samples were treated with varying concentrations of mifepristone for 48 hours. They found that mifepristone significantly decreased the viability of endometrial epithelial and stromal cells in adenomyosis and can induce their apoptosis as well [13]. This concentration-dependent inhibitory effect was most significantly seen with concentrations of mifepristone above 50 μmol/L at 48 hours. The same study showed that mifepristone demonstrated another dose-dependent relationship in the inhibition of the migration of ectopic endometrial and stromal cells. This finding is significant as the migratory nature of the patient’s endometrial and stromal cells is the pathogenesis behind adenomyosis [13].

Another study looked at the effect of mifepristone in combination with high-intensity focused ultrasound (HIFU) and levonorgestrel-releasing intrauterine system (LNG-IUS) in the treatment of adenomyosis [13]. Out of 123 patients, 34 patients were treated with HIFU alone, 29 patients were treated with HIFU combined with mifepristone, 10 patients with HIFU combined with LNG-IUS, and 50 patients with HIFU combined with mifepristone and LNG-IUS [13]. In the group treated with HIFU combined with mifepristone and LNG-IUS, the uterine volume was significantly reduced after treatment at 3, 6, 12, and 24 months compared to the previous treatment (p<0.05). Dysmenorrhea was measured using a visual analog score (VAS). In the combination group of mifepristone, HIFU, and LNG-IUS, VAS scores decreased from 80.82 ± 12.49 to 29.58 ± 9.29 at 24 months [13]. This was significantly lower than the three other treatment groups (p<0.05). The combination group of mifepristone, HIFU, and LNG-IUS also demonstrated statistically significant decreases in the menstrual volume and CA-125 serum markers [13]. Hemoglobin levels were not statistically different among the four treatment groups, but it is postulated that this could have been due to the fact that the patients who were anemic had been treated with different medications to improve their Hb aside from the trial medications [13]. 

Uterine leiomyomas are another gynecological condition that has been found to improve with the use of mifepristone as well. Insulin-like growth factor 1 (IGF-1) has been found to be overexpressed in leiomyomas [14]. This study showed that mifepristone inhibited the gene expression of IGF-1, and the reduction in symptoms correlated with a decrease in IGF-1 expression although the mechanism is not fully understood [14]. A meta-analysis studied the effects of mifepristone on uterine and leiomyoma volumes of 780 women from 11 randomized controlled trials. Mifepristone at doses from 2.5, 5, and 10 mg was found to effectively reduce uterine and leiomyoma volumes and alleviate leiomyoma symptoms at six months [6]. Pelvic pain, pelvic pressure, and dysmenorrhea were found to be alleviated after three months of treatment. Mifepristone also decreased the mean loss of blood during menstruation and a statistically significant increase in hemoglobin. No significant difference was found among varying dosages of 2.5, 5, and 10 mg other than increased frequency of hot flashes in patients of the 10 mg group. Another review investigated six clinical trials involving 166 women and the effects of 5-50 mg mifepristone for three to six months on leiomyomas [3]. The review demonstrated that daily treatment with all doses of mifepristone resulted in reductions in pelvic pain, pelvic pressure, dysmenorrhea, and uterine and leiomyoma volume size by 26-74%. Even doses of 2.5 mg of mifepristone resulted in significant improvement in the quality of life scores although there was little reduction in leiomyoma size at this dose [3]. This review also reported the rapid correction of uterine bleeding, amenorrhea, and increases in hemoglobin levels following treatment with 50 mg of mifepristone on alternating days. Even vaginal mifepristone has demonstrated efficacious results in the improvement of leiomyomas. In one such trial, the effects of daily 10 mg vaginal mifepristone were studied in 33 women from the ages of 30-53 [15]. Vaginal mifepristone significantly reduced leiomyoma volume and reduced the effects of symptoms on the patient’s quality of life as measured by the Uterine-Fibroid Symptoms Quality of Life questionnaire (UFS-QoL). It is important to note that the only significant side effect found in this review of trials was hot flashes at doses of mifepristone at 10 mg or more. Mifepristone was otherwise generally well tolerated with minimal if any adverse effects [15].

Discussion

Adenomyosis is a gynecologic condition that is characterized by the growth of endometrial cells into the myometrium, resulting in a globally enlarged uterus and an associated increase in CA-125 [16]. This marker is classically known to be an ovarian tumor marker; however, in this class, it reflects the increase in uterine glandular size. Although it is often labeled as a “benign” disease, it affects around 20% of reproductive-aged women. This condition can lead to dysmenorrhea, infertility, and menorrhagia in addition to detrimental effects on a patient’s mental health [16]. Despite 20% of affected patients being under the age of 40, the gold standard of treatment is a hysterectomy. Hysterectomies may often not be wanted by patients as it is an invasive surgery that comes with several potential complications of its own. It is important to note that due to the large percentage of patients with adenomyosis who are of reproductive age, hysterectomies may not be an appropriate standard method of treatment. To rob patients of their fertility without attempting medication therapy with mifepristone first is an act of injustice. Surgery alone comes with many complications and the possibility of recurrence. The ability of physicians to manage their patient’s pain and symptoms should be guided medically before surgical sterilization is considered. Many of these patients are forced to seek alternative non-invasive treatments instead of medication therapies to preserve their fertility.

HIFU and LNG-IUS are noninvasive therapies for adenomyosis that can be used in patients who refuse hysterectomies or for those who are not good candidates [16]. The pitfalls of these procedures include the fact that 20% of patients on HIFU alone end up relapsing, and LNG-IUS cannot be used in patients with a uterine size that is >12 weeks gestation or a uterine cavity depth that is >9 cm. Because adenomyosis is an estrogen-dependent disease, gonadotropin-releasing hormone agonists (GnRH-a) are also often used in combination with HIFU and LNG-US. Through the inhibition of the secretion of estrogen, GnRH-as facilitate reduced pelvic pain, reduced bleeding, and reduced uterine cavity size [16]. Reduction in cavity size is significant as this alone can lead to improved pain and reduced bleeding and allows patients to qualify for LNG-US where their previous uterine cavity size would have prevented their candidacy. Its current limitations include price (>$200/month), induction of premenopausal syndrome, and high rates of relapse following drug cessation [16]. Mifepristone offers a cheaper alternative (<$4/month) with significantly improved outcomes in reduced uterine cavity size, decreased dysmenorrhea pain scale score, and lower menstruation volume scores [16]. Mifepristone is also able to provide such results without the bone loss that is commonly associated with GnRH-analogs [3]. This is because mifepristone allows for serum estradiol to remain within the patient’s physiologic follicular phase range [3]. In addition, mifepristone is able to significantly reduce serum levels of CA-125 and improve hemoglobin levels in patients with menorrhagia. These reductions in CA-125 demonstrate marked reductions in the size of glands of the uterus of these patients. Through the reduction of cavity size, mifepristone can not only offer therapeutic relief but also allow patients to qualify for noninvasive LNG-US procedures, which can offer further therapeutic benefits. Patients should have the option to explore all potential medical therapies before opting for surgical correction.

Leiomyomas, or uterine fibroids, are another commonly encountered gynecologic condition and represent the most common benign tumors found in the female population. These benign smooth muscle tumors are estrogen-sensitive and can rarely develop into malignant leiomyosarcomas. Nearly 20-50% of patients with these fibroids experience symptoms, such as abnormal uterine bleeding (AUB), infertility, pelvic pain, and miscarriages [17]. Currently, the only treatment for this common condition is surgery. Two medications that are commonly used for preoperative reductions in leiomyoma size are mifepristone and enantone. Enantone is a gonadotropin-releasing hormone analog that has shown significant improvement in leiomyoma shrinkage, correction of anemia, and correction of AUB [17]. Through its MOA, however, enantone can lead to harmful adverse effects, such as menopausal symptoms and bone mineral loss. Using hormone supplementation to negate these side effects leads to reduced effectiveness of enantone in fibroid size reduction. Several studies have shown that progesterone plays a large role in the proliferation of leiomyoma growth [17]. Mifepristone, therefore, offers an effective alternate solution by producing the same results without enantone’s adverse effects. When comparing enantone to mifepristone, the two medications both resulted in statistically significant reductions in fibroid size, reduction in dysmenorrhea, reduction in non-menstrual abdominal pain, and increased Hgb/Hct/and RBC count despite differences in dosage [17]. However, mifepristone was able to maintain the patients’ premenopausal levels of estrogen, whereas patients on enantone were found to have estrogen levels of menopausal patients. Furthermore, patients who were treated with enantone also reported more adverse events compared to those in the mifepristone group [17]. Vaginal use of mifepristone has also been shown to significantly reduce leiomyoma size and improve symptoms of anemia while lowering systemic bioavailability of mifepristone [15]. Through its concentrated distribution to uterine tissue, vaginal mifepristone can lead to increased improvement in its clinical outcomes. Vaginal mifepristone showed statistically significant improvements in leiomyoma volume change, USF-QoL, and decreased bleeding intensity at the end of the three-month trial and three months after treatment [15]. For these reasons, mifepristone can be used effectively for conservative therapy in patients suffering from leiomyomas and should be considered a viable option for patients not wishing to undergo surgery. 

CD refers to hypercortisolism that is caused by pituitary adenomas, adrenal neoplasias, or paraneoplastic ACTH secretion. Hypercortisolism in these patients leads to the development of skin changes, HTN, obesity, insulin resistance, dyslipidemia, anovulation, skeletal disorders, and neuropsychiatric disorders [18]. Patients suffering from these conditions endure a severely decreased quality of life and increased morbidity and mortality. The syndromic nature of this disease prompts delayed diagnosis and further increases the mortality and morbidity of this population [18]. CS therefore necessitates effective and rapid treatment options to diminish harm and clinical burden. The current first-line treatment for CD is pituitary surgery despite its nearly ⅓ relapse rate within 10 years postoperatively [18]. In these patients and patients with recurrent CD, further treatment options are necessitated. These options include adrenal surgery, pituitary radiotherapy, or medication therapy. Radiotherapy further delays symptomatic relief as it usually takes years before excess cortisol levels are managed. It also carries the risk of the patient developing hypopituitarism due to subsequent pituitary damage [18]. While surgery of the adrenal glands can quickly achieve control of excess cortisol, it also carries a risk of permanent adrenal insufficiency. Medication therapy can be used preoperatively, postoperatively, and as adjunctive therapy to radiotherapy. These drug classes include somatostatin analogs, dopamine agonists, and adrenal steroidogenesis inhibitors [18]. The most commonly used medication is the adrenal steroidogenesis inhibitor ketoconazole. While it has been proven to be effective and rapid in its success, doses may need to be frequently increased due to the cortisol blockade that occurs in CD patients [8]. In fact, due to the hormonal imbalances in CD patients, many medications often have to be dose adjusted to achieve therapeutic effect. It is also important to note that many of the medications that are used are not easily tolerated when doses are increased or adjusted frequently. The use of mifepristone has demonstrated statistically significant results in weight reduction, insulin resistance, depression, HTN, and quality of life in CD patients [10]. Furthermore, mifepristone can also be used effectively in patients experiencing cortisol-induced psychosis during acute exacerbations of hypercortisolism. While not included in the classes of more commonly used drugs for CD, mifepristone has been approved by the FDA for the treatment of CD when associated with disorders of glucose metabolism. This is undoubtedly due to the stigmatization of mifepristone and the subsequent reluctance of clinicians to incorporate it into their treatment plans.

Neuropsychiatric disorders have been investigated for their associations with dysregulations of the hypothalamic-pituitary-adrenal axis (HPA) and increases in cortisol levels. Studies have shown that patients suffering from depression, schizophrenia, and psychotic depression have elevated levels of cortisol and increased activity of their HPA [19]. The role of cortisol in psychiatric disorders is evidenced by the adverse psychiatric effects that patients can develop in response to exogenous glucocorticoid use through subsequent increases in cortisol. These include delirium, depression, mania, or psychosis. When functioning normally, HPA activity and cortisol secretion are maintained through sensitive negative feedback systems involving glucocorticoid receptors (GCRs) and mineralocorticoid receptors (MCR) [19]. At low doses, cortisol preferentially binds to MCR. As cortisol levels rise, it begins to bind to GCR and thereby initiates the negative feedback loop. Antipsychotics that are typically used work by reducing cortisol levels. Mifepristone, when dosed at >200 mg/day, selectively binds only to GCR and has no effect on MCR [19]. Through its sole inhibition of GCR, it ensures that normal cortisol homeostasis is maintained while ensuring that excess high levels of cortisol are blocked. This was evidenced by the statistically significant correlation between rising plasma concentrations of mifepristone and improvement of psychotic symptoms [20].

The hippocampus is a region of the temporal lobe that is most notably recognized for its role in learning and memory. Further studies have shown correlations between hippocampal atrophy and patients with severe depression, PTSD, and schizophrenia. It is postulated that this hippocampal atrophy leads to persistently high levels of cortisol, worsening these patient’s psychiatric symptoms. Administration of mifepristone to patients with combat-related PTSD demonstrated significant benefits in quality of life and psychiatric improvement. Psychotic major depression is another psychiatric condition that affects around 20% of patients with major depression [7]. When mifepristone was used to treat psychotic depression, patients were able to achieve rapid antipsychotic effects that lasted for weeks after the medication therapy ended. It should be noted that patients suffering from PMD generally have increased cortisol levels even with standard antidepressant therapy alone [7]. Some patients are even unresponsive to electroconvulsive therapy. The ability of patients suffering from psychotic depression to achieve rapid relief is imperative as these patients are more susceptible to suicidal ideation, especially during an episode of psychosis [7]. Bipolar disorder is another mood disorder that has been found to be associated with high levels of cortisol, dysfunction of the HPA axis, and GR dysfunction. Several neuroendocrine studies demonstrated that around 43% of bipolar patients with depression were also dexamethasone-suppression-test (DST) nonsuppressors [7]. Further studies found that bipolar patients suffering through relapse and recovery had abnormal dexamethasone/corticotropin-releasing hormone (dex/CRH) test results [21]. These abnormal (dex/CRH) findings were also seen in healthy patients who had certain genetic predispositions for mood disorders [21]. Regarding these HPA dysfunctions, GR has been implicated in being an important modulator of neurocognitive function and mood. This can be evidenced through research findings that report increased GR number and GR binding in brain tissue following the administration of antidepressants in depressed patients [21].

Mifepristone’s unique advantage is that its selective role as a GR antagonist was also found to increase both MR and GR binding in the frontal cortex. In fact, data from Young et al. [21] reveals significant improvement in frontal cortex functioning following clinical mifepristone trials. These results were seen through improvements in spatial working memory function and reductions in the HDRS17 and MADRS. They also demonstrated significant improvement in verbal fluency from baseline. These improvements in neurocognitive functioning were measured when the subjects’ mood was similar to their baseline or did not vary when compared to the placebo group [21]. This key finding suggests that improvements in neurocognitive functioning were not solely related to improvements in mood or depression. Mifepristone achieves these improvements in neurocognitive function through its selective activity towards GR within the frontal cortex. Furthermore, patients are also able to achieve symptomatic improvement two weeks after the initiation of treatment [21]. The rapid nature of mifepristone adds further clinical benefit as classic bipolar treatments take longer to achieve therapy and the fact that treatment plans for patients with bipolar disorder are tricky to individualize. Other commonly known psychiatric disorders are treated with antipsychotics. While these medications often come with a large array of adverse effects, weight gain, metabolic derangements, and glucose intolerance have been a few of the more frequently reported negative effects. While the exact cause of the weight gain is unknown, mifepristone was shown to significantly reduce weight gain in patients when taken alongside risperidone or olanzapine [21]. As discussed previously, mifepristone also has the ability to significantly improve insulin resistance, thereby further improving the AE patients may experience on antipsychotics. Therefore, through mifepristone’s selective activity as a GCR antagonist, it has immense potential as a psychiatric therapeutic agent.

## Conclusions

Mifepristone is a synthetic steroid that has immense potential to provide symptomatic relief in patients suffering from a wide array of complicated diseases. Prednisone, dexamethasone, and anabolic steroids are also synthetic steroids that are commonly used. Despite being a part of the same class as mifepristone, none of these medications fall under as much legal, political, and social duress as mifepristone. This is in spite of the fact that mifepristone has been proven to have an incredible safety profile since its introduction to the public in the 1980s. In fact, its mortality rate is significantly lower than that of Tylenol, NSAIDs, penicillin, and phosphodiesterase inhibitors. While further research is certainly needed, its involvement in politics has unfortunately led to the willful ignorance of its medical potential despite its evidence-based safety profile and efficacy.
